# Identification and characterization of a novel Epstein-Barr Virus-encoded circular RNA from *LMP-2* Gene

**DOI:** 10.1038/s41598-021-93781-w

**Published:** 2021-07-13

**Authors:** Ke-En Tan, Wei Lun Ng, Georgi K. Marinov, Ken Hung-On Yu, Lu Ping Tan, Ee Shan Liau, Sook Yan Goh, Kok Siong Yeo, Kevin Y. Yip, Kwok-Wai Lo, Alan Soo-Beng Khoo, Lee-Fah Yap, Chee-Kwee Ea, Yat-Yuen Lim

**Affiliations:** 1 Institute of Biological Sciences, Faculty of Science, Universiti Malaya, 50603 Kuala Lumpur, Malaysia; 2 Department of Biology, Indiana University Bloomington, Bloomington, IN 47405-7005 USA; 3 Department of Computer Science and Engineering, The Chinese University of Hong Kong, Shatin, New Territories Hong Kong; 4 Department of Anatomical and Cellular Pathology, The Chinese University of Hong Kong, Shatin, New Territories Hong Kong; 5 Molecular Pathology Unit, Cancer Research Centre, Institute for Medical Research, National Institutes of Health, Ministry of Health Malaysia, 40170 Selangor, Malaysia; 6 Hong Kong Bioinformatics Centre, The Chinese University of Hong Kong, Shatin, New Territories Hong Kong; 7 Hong Kong Institute of Diabetes and Obesity, The Chinese University of Hong Kong, Shatin, New Territories Hong Kong; 8 Department of Oral & Craniofacial Sciences, Faculty of Dentistry, Universiti Malaya, 50603 Kuala Lumpur, Malaysia; 9 Oral Cancer Research and Coordinating Centre, University of Malaya, 50603 Kuala Lumpur, Malaysia; 10 Present Address: Department of Genetics, Stanford University, Stanford, CA 94305 USA; 11 Present Address: Programme in Emerging Infectious Diseases, Duke-NUS Medical School, Singapore, Singapore; 12 Present Address: Institute of Molecular Biology, Academia Sinica, Taipei, Taiwan; 13 Present Address: Department of Biochemistry and Molecular Biology, Mayo Clinic College of Medicine, Mayo Clinic Cancer Center, Rochester, MN 55902 USA; 14 Present Address: Department of Molecular Biology, University of Texas Southwestern Medical Center, Dallas, TX 75390-9148 USA

**Keywords:** Non-coding RNAs, Virology, Tumour virus infections

## Abstract

Epstein-Barr virus (EBV) has been recently found to generate novel circular RNAs (circRNAs) through backsplicing. However, comprehensive catalogs of EBV circRNAs in other cell lines and their functional characterization are still lacking. In this study, we have identified a list of putative EBV circRNAs in GM12878, an EBV-transformed lymphoblastoid cell line, with a significant majority encoded from the EBV latent genes. A novel EBV circRNA derived from the exon 5 of *LMP-2* gene which exhibited highest prevalence, was further validated using RNase R assay and Sanger sequencing. This circRNA, which we term circ*LMP-2_e5*, can be universally detected in a panel of EBV-positive cell lines modelling different latency programs. It ranges from lower expression in nasopharyngeal carcinoma (NPC) cells to higher expression in B cells, and is localized to both the cytoplasm and the nucleus. We provide evidence that circ*LMP-2_e5* is expressed concomitantly with its cognate linear *LMP-2* RNA upon EBV lytic reactivation, and may be produced as a result of exon skipping, with its circularization possibly occurring without the involvement of *cis* elements in the short flanking introns. Furthermore, we show that circ*LMP-2_e5* is not involved in regulating cell proliferation, host innate immune response, its linear parental transcripts, or EBV lytic reactivation. Taken together, our study expands the current repertoire of putative EBV circRNAs, broadens our understanding of the biology of EBV circRNAs, and lays the foundation for further investigation of their function in the EBV life cycle and disease development.

## Introduction

Epstein-Barr virus (EBV) is a lymphotropic DNA herpesvirus that infects approximately 95% of the world’s population^1^. EBV can cause infectious mononucleosis^2^ in children and is associated with various malignancies in lymphocytes and epithelial cells such as Burkitt lymphoma (BL), NK/T cell lymphoma (NKTCL), gastric carcinoma (GC) and NPC^3^. EBV can establish two types of infection in cells: latent and lytic. EBV remains latent in infected memory B-cells^4^ and with periodic reactivation of lytic replication, the salivary glands within the nasopharynx or throat epithelium could be a source for EBV infection to the epithelial cells^5,6^. During the latency period, the EBV genome exists as a circular episome in the nucleus that is maintained via a unique replication mechanism^7^. Upon reactivation into the lytic cycle, EBV briefly passes through three consecutive phases—immediate early (IE), early (E), and late (L)—to produce infectious virions^8^. Importantly, previous studies have shown that both EBV coding and non-coding genes from latent and lytic cycles contribute to the pathogenesis of EBV-associated diseases^8,9^.

EBV has recently been demonstrated to express a diverse repertoire of circular RNAs (circRNAs), an intriguing class of non-coding RNA (ncRNA) involved in multiple biological processes. The investigation of the prevalence and biological roles of circRNAs has only begun in recent years thanks to advances in high-throughput RNA sequencing techniques and of computational methods for their detection. CircRNAs are formed through a unique mechanism known as backsplicing, whereby the upstream 3′ splice acceptor is covalently joined to the downstream 5′ splice donor. Due to the absence of free termini in the circular structure, circRNA is resistant to hydrolysis by numerous cellular exonucleases such as RNase R^10^. CircRNAs can be detected in a wide diversity of species across all examined eukaryote clades, with thousands of circRNAs reported to be highly expressed in tissue- or developmental stage-specific manners^11^. CircRNAs of different lengths can be derived from exons (ecircRNAs), introns (ciRNAs), or both (EIcircRNAs), with ecircRNAs being the most common type. Several biological functions of circRNAs have been demonstrated and proposed, including being miRNA sponges^12^, regulation of their parental gene expression through *cis*- or *trans*-actions^13^, serving as mRNA traps^14^, protein binding platforms^15^, and even coding for proteins^16^.

Multiple EBV circRNAs, with the majority in the form of ecircRNAs consisting of one or more exons, and a few including intronic regions, were reported recently^17,18^. The abundance of EBV circRNAs is affected by the EBV infection state. Lytic reactivation in the Akata-BL cell line specifically, leads to the production of multiple different alternative backspliced isoforms that are not seen in other cell lines. Although most of the EBV circRNAs are of low abundance, some are expressed at levels comparable to cellular RNA levels such as circ*BHLF1* and circ*RPMS1_E4_E3a*^17^. In fact, circ*RPMS1_E4_E3a* was reported to be one of the most abundant circRNAs found in EBV-positive cell lines of different latencies, NPC and GC patient-derived xenografts, as well as in patient samples from NPC, GC and post-transplant lymphoproliferative disorders (PTLDs)^17–19^. A few EBV circRNAs are even conserved across members of Gammaherpesviridae. For example, circ*RPMS1_E5_E3a* in the rhesus macaque lyphocrytovirus (rLCV) is an ortholog to EBV circ*RPMS1_E4_E3a*^20^. In addition, EBV circRNAs can localize to either the cytoplasm, the nucleus or both. However, to date, the full catalogue of EBV transcribed circRNAs in other EBV-positive cell lines and disease backgrounds remains to be fully explored. Notably, limited functions have been ascribed to these EBV-derived circRNAs, with only circRNAs from the *RPMS1* and *LMP-2A* genes having been investigated in detail^19,21,22^.

Here we report an in silico analysis of putative EBV circRNAs expressed in the GM12878 cells, an EBV-positive lymphoblastoid cell line with type III latency, and investigate the role of EBV circRNA in regulating host or viral genes and/or signaling pathways. Among the putative EBV circRNAs, the *bona fide* experimentally validated EBV circRNA with the highest read count in the latent state, circ*LMP-2_e5*, was chosen for further study. We characterize the expression of circ*LMP-2_e5* across a panel of cell lines of different EBV latencies as well as in the lytic state, and investigate its biogenesis and potential functions.

## Results

### Identification of EBV circRNAs in GM12878 RNA-sequencing (RNA-seq) datasets

To comprehensively identify EBV-derived circRNAs, the GM12878 RNA-seq datasets made available by the ENCODE consortium were re-analyzed using two different algorithms, psirc^23^ and find_circ^24^. GM12878 is a lymphoblastoid cell line (LCL) that exhibits EBV latency III, for which RNA-seq datasets generated from various subcellular poly-A and non-poly-A enriched fractions are available, which makes it ideal for generating a full catalogue of EBV circRNAs.

**Figure 1:**
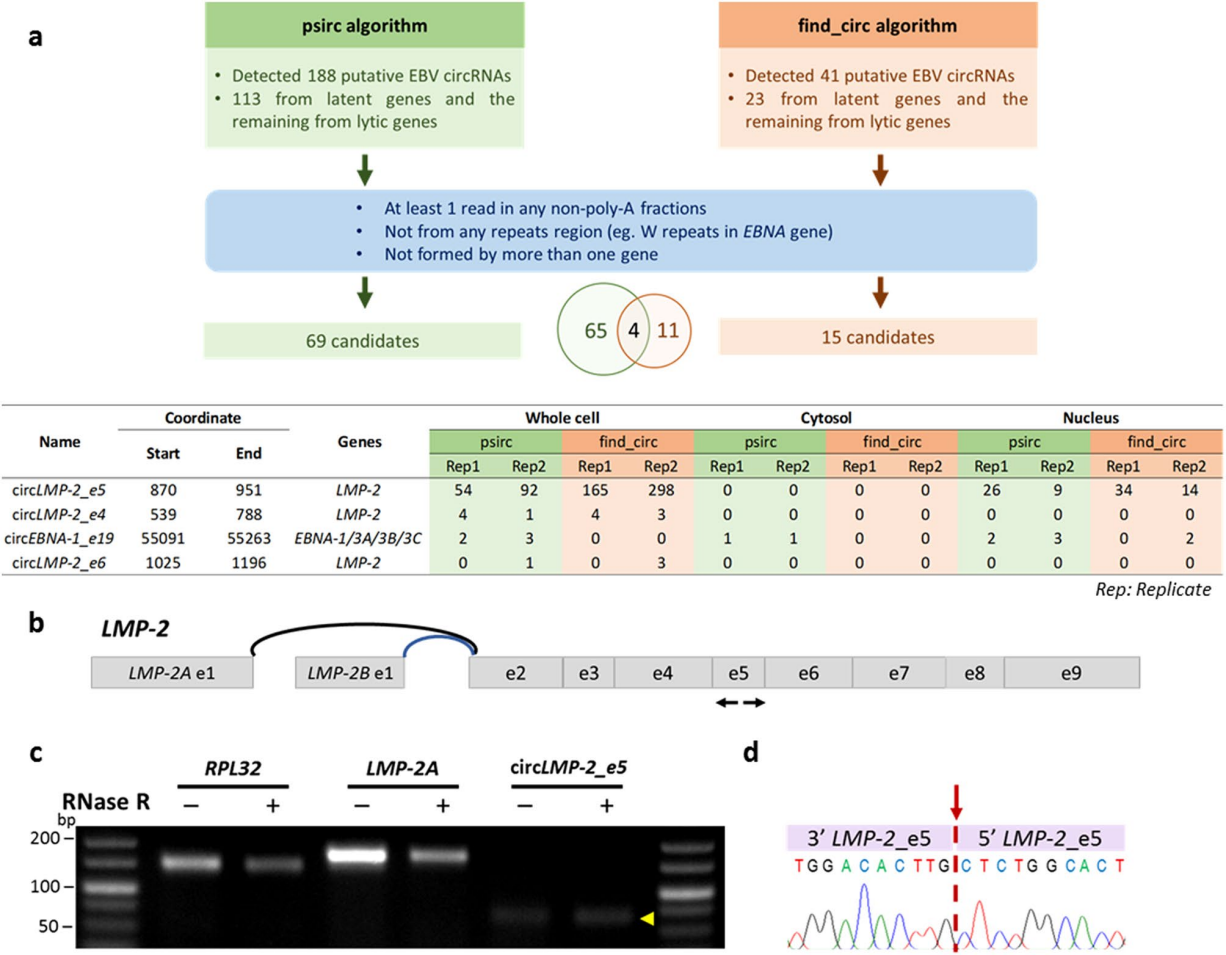
Screening and validation of putative EBV circRNAs in GM12878 cell line. (**a**) Schematic summary demonstrating the strategy for identification and filtering of putative EBV circRNAs from RNA-seq datasets. The table shows the location, exons involved and read counts for overlapping putative EBV circRNAs identified from non-poly-A subcellular fractions by both algorithms, psirc and find_circ. (**b**) Schematic representation of divergent primer pairs used for the detection of BSJ of circ*LMP-2*_e5 identified by the two algorithms. Black arrows indicate the position of divergent primer pairs used. (**c**) RNase R resistance of circ*LMP-2*_e5 in the GM12878 cell line, as indicated by the yellow arrowhead. The full-length gel image is shown in Fig. S6. (**d**) The expected PCR product was Sanger sequenced and the BSJ of circ*LMP-2*_*e5* was confirmed, as indicated by the red downward arrow.

Searching for backspliced junctions (BSJs), a total of 188 putative circRNAs encoded by EBV were detected by the psirc algorithm, whereas find_circ identified 41 EBV circRNA candidates. Based on the EBV BSJs detected by psirc and find_circ algorithms, 60% (133/188) and 56% (23/41) of putative circRNAs are produced from EBV latent genes, respectively (Fig. 1a, Tables S1–S2). Sixty-nine and fifteen putative circRNAs identified from each algorithm fulfilled the criteria of BSJ reads ≥ 1 in the non-poly-A fraction and were not derived from any repetitive regions or from more than one gene. For example, a majority of the EBV circRNAs candidates were encoded by *EBNA*s and most of them fall within the W1–W2 repeats region, with only one exception (circ*EBNA-1_e19*). Putative EBV circRNAs derived from the W1–W2 repeats region of *EBNA*s may be an artifact of the exon concatemers in the linear mRNA leading to the false prediction of cirRNAs, and were therefore excluded from further analysis. Likewise, candidate circRNAs from the *IR1* locus were also excluded. There was only one circRNA candidate from *LMP-1,* while the rest were from *LMP-2*. Putative EBV circRNAs from *LMP-2* are all originated from the common regions shared between both *LMP-2* isoforms (*LMP-2A* and *LMP-2B*). Amongst all the EBV cirRNA candidates detected, four putative EBV circRNAs were identified in both algorithms (Fig. 1a). The novel EBV circRNA derived from exon 5 of the *LMP-2* gene (termed as circ*LMP-2_e5*) was chosen out of these four putative EBV circRNAs for further study as it has the highest BSJ read counts in non-poly-A fractions with no reads in poly-A fraction in both bioinformatics analysis (Fig. 1a).

To rule out the possibility that these circRNAs are formed due to *trans*-splicing or genomic rearrangement, circ*LMP-2_e5* was validated using RNase R treatment as well as by PCR using divergent primers (Fig. 1b). RNase R is an exoribonuclease that specifically degrades linear, but not circular or lariat RNAs^25^. As shown in Fig. 1c, circ*LMP-2_e5* was detected in GM12878 cells and further enriched upon RNase R treatment. As expected, linear *RPL32* and *LMP-2A* were substantially decreased in abundance after RNase R treatment (Fig. 1c). Sanger sequencing of the expected amplicon confirmed the BSJ of circ*LMP-2_e5* (Fig. 1d), confirming it as a *bona fide* EBV circRNA.

### Characterization of circ*LMP-2_e5* in EBV-positive cell lines

**Figure 2:**
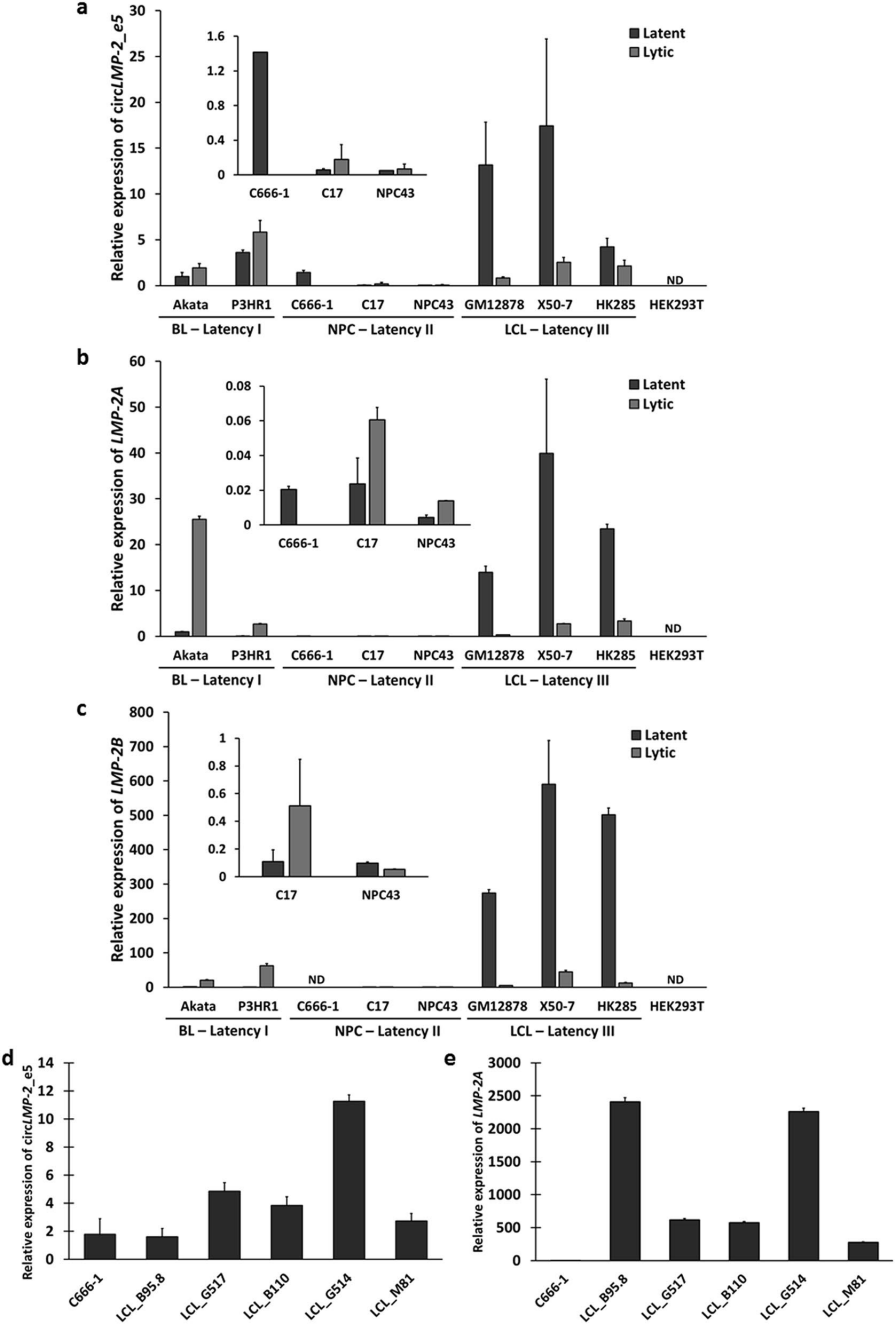
RT-qPCR analysis of linear *LMP-2* and circ*LMP-2*_e5 in various EBV-positive cell lines. Expression of (**a**) circ*LMP-2_e5,* and linear (**b**) *LMP-2A* and (**c**) *LMP-2B* in EBV-positive cell lines with different latency programs in latent and lytic states. Akata was reactivated using Goat (Fab)_2_ fragment anti-human immunoglobulin G (IgG) for 24 h while C17 was reactivated by transfection with BZLF1-expressing plasmid p509 for 72 h using X-tremeGene HP DNA transfection reagent. The remaining cell lines were induced into lytic cycle using TPA and SB for 72 h except for NPC43 which was induced for 48 h. C666-1 cells were unable to be reactivated and this is consistent with previous reports of the abortive nature of the lytic reactivation in these cells. Human embryonic kidney cell line (HEK293T), an EBV-negative cell line was used as a negative control. Data was normalized to *UBC* and relative to gene expression in Akata cells. Data represents the mean ± SD of two independent experiments. (**d**, **e**) Expression of circ*LMP-2_e5* and linear *LMP-2A* in newly generated LCLs. Data was normalized to *UBC* and relative to gene expression in C666-1 cells. Data represents the mean ± SD of two independent experiments. *ND* not-detected*.*

Analysis of ENCODE RNA-seq datasets by Salzman and co-workers previously highlighted the cell-type specificity of human circRNA expression as one of the key features of circRNAs^26^. To examine whether circ*LMP-2_e5* is limited to GM12878 cells only or could be detected in other EBV-positive cells as well, we characterized circ*LMP-2_e5* expression in a series of cell lines that represent different types of EBV latency status, including both B lymphocytes and epithelial cells. Whereas Akata and P3HR1 cells are EBV-positive BL cell lines that represent latency I, C666-1, C17 and NPC43 are EBV-positive NPC cell lines that represent latency II, and the EBV-transformed LCLs GM12878, X50-7 and HK285 are of type III latency. As illustrated in Fig. 2a, circ*LMP-2_e5* was detected in all of the cell lines with the LCLs having the highest expression in latent state as compare to BL and NPC cell lines. To determine the expression of circ*LMP-2_e5* in the lytic state, we induced lytic reactivation in the different cell lines. Level of induction for each system, indicated by expression of *BZLF1*, *BMRF1* and *gp350*, are shown in Fig. S1. Upon lytic reactivation, we found that circ*LMP-2_e5* expression increased in latency I and II cell lines but showed reduction in latency III cell lines (Fig. 2a). P3HR1 cells have the highest circ*LMP-2_e5* expression upon lytic reactivation while expression of circ*LMP-2_e5* in EBV-positive NPC cell lines remain lowest in lytic states. These results suggest that B cells express more circ*LMP-2_e5* as compared to NPC cells. Moreover, the expression of circ*LMP-2_e5* correlates positively with linear *LMP-2* expression upon lytic reactivation as shown in Fig. 2b and c.

In addition to the existing EBV-positive cell lines, we also examined the expression of circ*LMP-2_e5* in newly developed LCLs generated by infecting healthy donor B cells with EBVs derived from infectious mononucleosis patient (B95.8) and NPC cases (M81, G517, B110 and G514). As expected, all newly generated LCLs expressed both *LMP-2A* and circ*LMP-2_e5* (Fig. 2d,e)*.* Interestingly, LCLs generated by NPC-derived EBV generally had higher expression of circ*LMP-2_e5* as compared to LCLs generated by B95.8. LCL_G514 harbored the highest expression of circ*LMP-2_e5* whereas LCL_B95.8 had the lowest expression of circ*LMP-2_e5*. Together, this result suggests that circ*LMP-2_*e5 expression is not unique to the existing EBV-positive cell lines but also in all newly generated LCLs.

**Figure 3:**
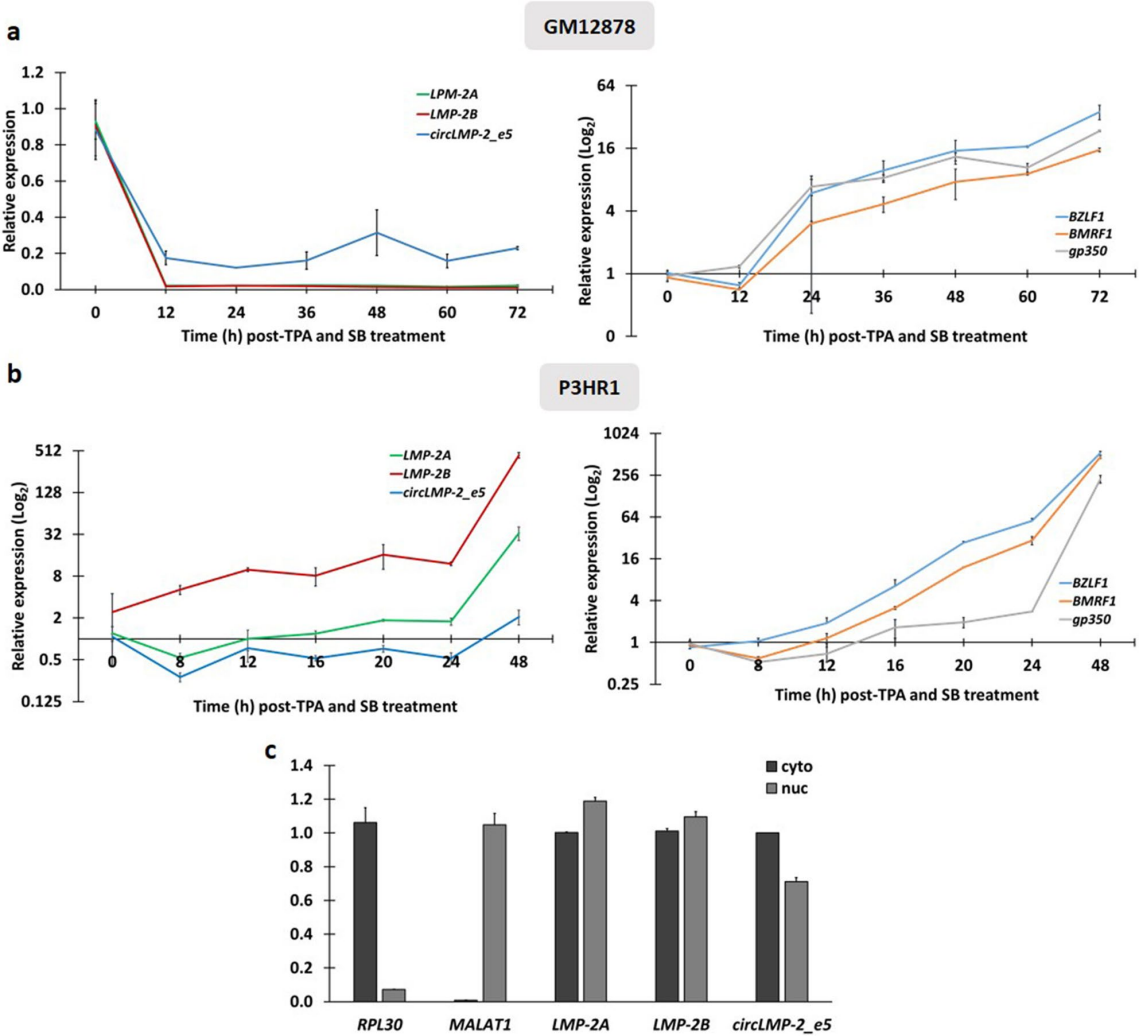
Temporal and spatial expression of linear *LMP-2* and circ*LMP-2_e5*. (**a**, **b**) Kinetic expression of EBV linear *LMP-2* and circ*LMP-2*_e5, as well as EBV lytic genes in (**a**) GM12878 and (**b**) P3HR1 cells were carried out for the indicated time period using RT-qPCR. Data was normalized to *RPL32*/*UBC* and represents the mean ± SD of two independent experiments. (**c**) RT-qPCR analysis using junction-specific divergent primers showed *LMP-2* and circ*LMP-2*_e5 were located in both nucleus and cytoplasm of GM12878 cells. Data was normalized to the RNA yield ratio and represents the mean ± SD of two independent experiments.

To further investigate the expression dynamics of circ*LMP-2*_e5 in a more detail manner, a time-course analysis of the expression of EBV lytic genes, linear *LMP-2* and circ*LMP-2*_e5 was carried out in GM12878 and P3HR1 cells (Fig. 3a and d). GM12878 and P3HR1 cells were chosen as these two cell lines are B cells which express higher amount of circ*LMP-2_e5* and show different circ*LMP-2_e5* expression patterns upon lytic reactivation, whereby circ*LMP-2_e5* expression is reduced in GM12878 cells, but increases in P3HR1 cells (Fig. 2a). In GM12878 cells, upon entering the lytic state after 12 h of TPA and SB treatment (Fig. 3a, right panel), both circ*LMP-2_e5* and its linear *LMP-2* expression show a large decline (Fig. 3a, left panel). In contrast, circ*LMP-2_e5* and linear *LMP-2* only showed an obvious increase in expression at 48 h post lytic reactivation in P3HR1 cells, which is the late phase of lytic reactivation (Fig. 3b, left panel). This kinetic expression data further supports the expression pattern of circ*LMP-2_e5* is associated with the expression of linear *LMP-2* in GM12878 and P3HR1 cells.

Cellular localization of circRNAs may provide some hints on their biological functions, for example nuclear-retained circRNAs are predicted to have a role in transcription regulation, whereas circRNAs that are predominantly found in cytoplasm are more likely to be involved in post-transcriptional gene regulation. To understand the physiological role of circ*LMP-2_e5*, the subcellular localization of circ*LMP-2_e5* in GM12878 cells was determined. The cytoplasmic *RPL30* and nuclear *MALAT1* transcripts were used as controls to indicate the purity of cytoplasmic and nuclear fractions, respectively. As expected, *RPL30* was predominantly enriched in the cytoplasmic fraction whereas *MALAT1* was enriched in the nuclear fraction (Fig. 3c). Intriguingly, circ*LMP-2_e5* was found in both cytoplasmic and nuclear fractions, which differs from the pattern observed in ENCODE datasets, in which circ*LMP-2_e5* is seen only in nuclear fraction. Nonetheless, the subcellular localization pattern of circ*LMP-2_e5* is similar to linear *LMP-2A* and *LMP-2B,* suggesting that circ*LMP-2_e5* may exhibit different functionalities by exerting distinct regulatory effects at different cellular compartments^27,28^.

### Exon 5-skipped LMP-2 variant and biogenesis of circ*LMP-2_e5*

**Figure 4:**
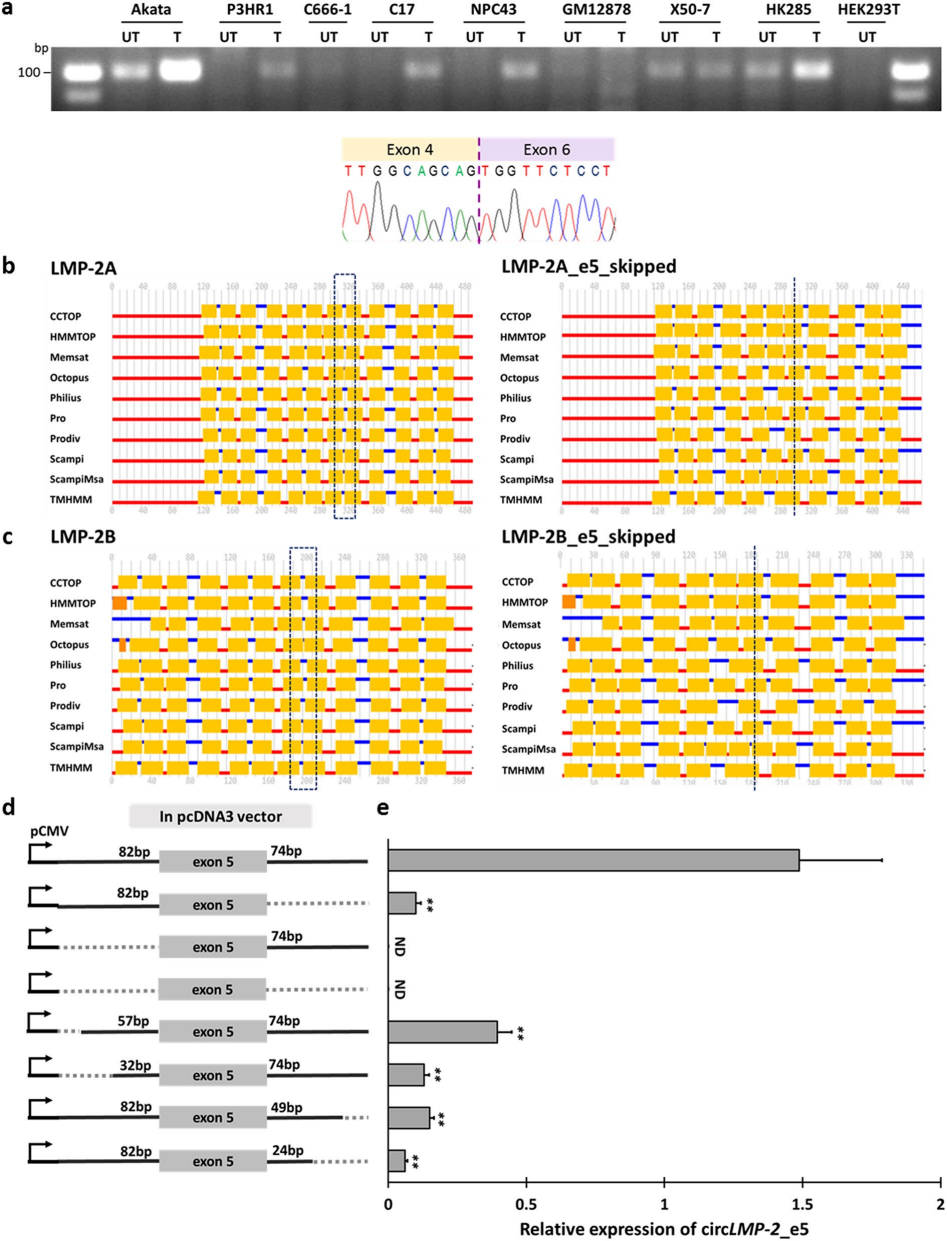
Exon 5-skipped LMP-2 splice variant and biogenesis of circ*LMP-2_*e5 and matched cognate exon 5 skipping in *LMP-2* splice variant. (**a**) The *LMP-2* splice variant with *LMP-2* exon 5-skipped was detected in EBV-positive cell lines with different latency programs in latent (UT) and lytic (T) states. The full-length gel image is shown in Fig. S6. The *LMP-2* splice variant with exon 4 and 6 fused-amplicon was validated using Sanger sequencing. (b-c) Transmembrane topology prediction of (**b**) LMP-2A and (**c**) LMP-2B with or without *LMP-2* exon 5-skipping using multiple prediction tools. The black dotted box indicates amino acids (position 308 to 333 and 188 to 214 for LMP-2A and LMP-2B respectively) that are encoded by exon 5 of *LMP-2* whereas the black dotted line indicates the amino acid position where the exon 4 and exon 6 have joined in exon 5-skipped *LMP-2A* or *LMP-2B*. (**d**) Schematic representation of pcDNA3 constructs with wild type and truncated introns upstream and downstream of *LMP-2* exon 5 used to test circ*LMP-2_e5* expression. Black solid lines and grey boxes represent introns and exon respectively, whereas light grey dotted lines indicates the deleted region of the introns. Each construct was transfected into HONE (EBV negative NPC) cells using Roche X-tremeGENE HP transfection reagent. (**e**) Relative expression of circ*LMP-2_e5* from different truncated intron constructs. Data represents mean ± SEM of four independent experiments. Significant *p* values [≤ 0.05 (*) and ≤ 0.01 (**)] as determined by Student’s T-test are indicated. *ND* not-detected*.*

Exon circularization events are positively correlated with cognate linear mRNA exon-skipping either through formation of lariat intermediates or through direct backsplicing^29–32^. To assess whether circ*LMP-2_e5* formation might be a by-product of exon skipping in the cognate *LMP-2* transcript, we designed primer pairs to specifically detect *LMP-2* splice variants with exon 5-skipped. Using a common primer set that could amplify both exon 5 inclusion and skipped isoforms, we were able to detect the normal *LMP-2* transcript together with weak amplicons that may correspond to *LMP-2* splice variant with exon 5-skipped in latent GM12878 and P3HR1 cells, as well as in the lytic reactivated P3HR1 cells using RT-PCR followed by agarose gel electrophoresis (Fig. S2). To reliably detect *LMP-2* splice variants with exon 5-skipped, we designed a reverse primer that spans the junction between exon 4 and 6, and successfully validated the splice variant and confirmed the fusion of exon 4 and 6 with Sanger sequencing (Fig. 4a). Moreover, the exon 5-skipped *LMP-2* splice variant can be detected in various EBV-positive cell lines with different latency programs. In general, its expression patterns were similar to the expression pattern of circ*LMP-2_e5* in latent and lytic states, except for latency III cell lines, which showed a different trend with either an unchanged or reverse pattern in X50-7 and HK285 cells respectively. These data suggest that exon skipping might give rise to circ*LMP-2_e5* formation in latency I and II cell lines.

LMP-2 is a hydrophobic membrane protein with two isoforms, LMP-2A and LMP-2B^33^. The LMP-2A primary amino acid sequence contains 119 amino acids at the amino terminus that is not shared by LMP-2B, while the rest of its amino acid sequence is shared between both isoforms and forms twelve hydrophobic domains of at least 16 amino acids, each of which traverses the plasma membrane followed by a 27 amino acid carboxyl terminal domain. To determine the effect of exon 5 skipping on LMP-2 protein structure, we compared the transmembrane domains for LMP-2A and LMP-2B with or without *LMP-2* exon 5-skipping using multiple transmembrane topology prediction tools. Skipping of *LMP-2* exon 5 makes the protein shorter by 27 amino acids and leads to the loss and/or fusion of the transmembrane domains 7 and 8 of both LMP-2 proteins as showed in Fig. 4b and c. As a result, the carboxyl terminal domain of the exon 5 skipped LMP-2 splice variant may be localized to a different side of the plasma membrane.

Next, we analyzed the flanking introns of *LMP-2* exon 5 for tell-tale signs of circRNA processing. Recent studies have demonstrated that circularizable exons are flanked by long introns^34,35^. However, the flanking upstream and downstream sequences of circ*LMP-2_e5* are only 82 bp and 74 bp, i.e. they are relatively short. We generated a pcDNA3 construct consist of *LMP-2* exon 5 along with its flanking upstream and downstream introns and introduced the construct into an EBV-negative NPC cell line, HONE1 cells (Fig. 4d). Expression of circ*LMP-2_e5* was determined using qRT-PCR 48 h post-transfection. We found that the BSJ of circ*LMP-2_e5* can be detected in HONE1 cells (Fig. 4e) suggesting that the short flanking introns are sufficient to generate circ*LMP-2_e5*. Although it has been reported that short sequences (as small as 30 to 40 nucleotides) are sufficient to facilitate circRNA biogenesis, those require RNA pairing across flanking introns to enable RNA duplex formation to efficiently promote exon circularization^31^. Upon sequence analysis, no such *cis*-elements, such as repetitive *Alu* elements or non-repetitive inverted complementary sequences, were found in the flanking introns (data not shown), suggesting formation of circ*LMP-2_e5* is not promoted by such mechanism. To determine which intronic regions are essential for *LMP-2* exon 5 circularization, a series of pcDNA3 constructs with truncated upstream and downstream introns of *LMP-2* exon 5 (Fig. 4d) were constructed and tested in HONE cells. As expected, deletion of both upstream and downstream introns prevents the circularization of *LMP-2* exon 5 (Fig. 4e). Intriguingly, only deletion of the upstream intron and not the downstream intron completely abolished the circularization of *LMP-2* exon 5. The latter retained minute expression of circ*LMP-2_e5* (~ 10%). These data suggest that the upstream intron is more essential for *LMP-2* exon 5 circularization compared to the downstream intron. Whereas deletion of 25 bp and 50 bp of upstream intron caused 60% and 87% reduction in circ*LMP-2_e5* expression, respectively, the same deletion of the downstream intron caused 85% and 94% reduction in circ*LMP-2_e5* expression. These results suggest canonical backsplicing by spliceosome or the presence of additional mechanisms of exon circularization for *bona fide* circ*LMP-2_e5* biogenesis, which does not rely on long introns nor any repetitive or inverted complementary sequences.

### Functional characterization of circ*LMP-2*_*e5*

**Figure 5:**
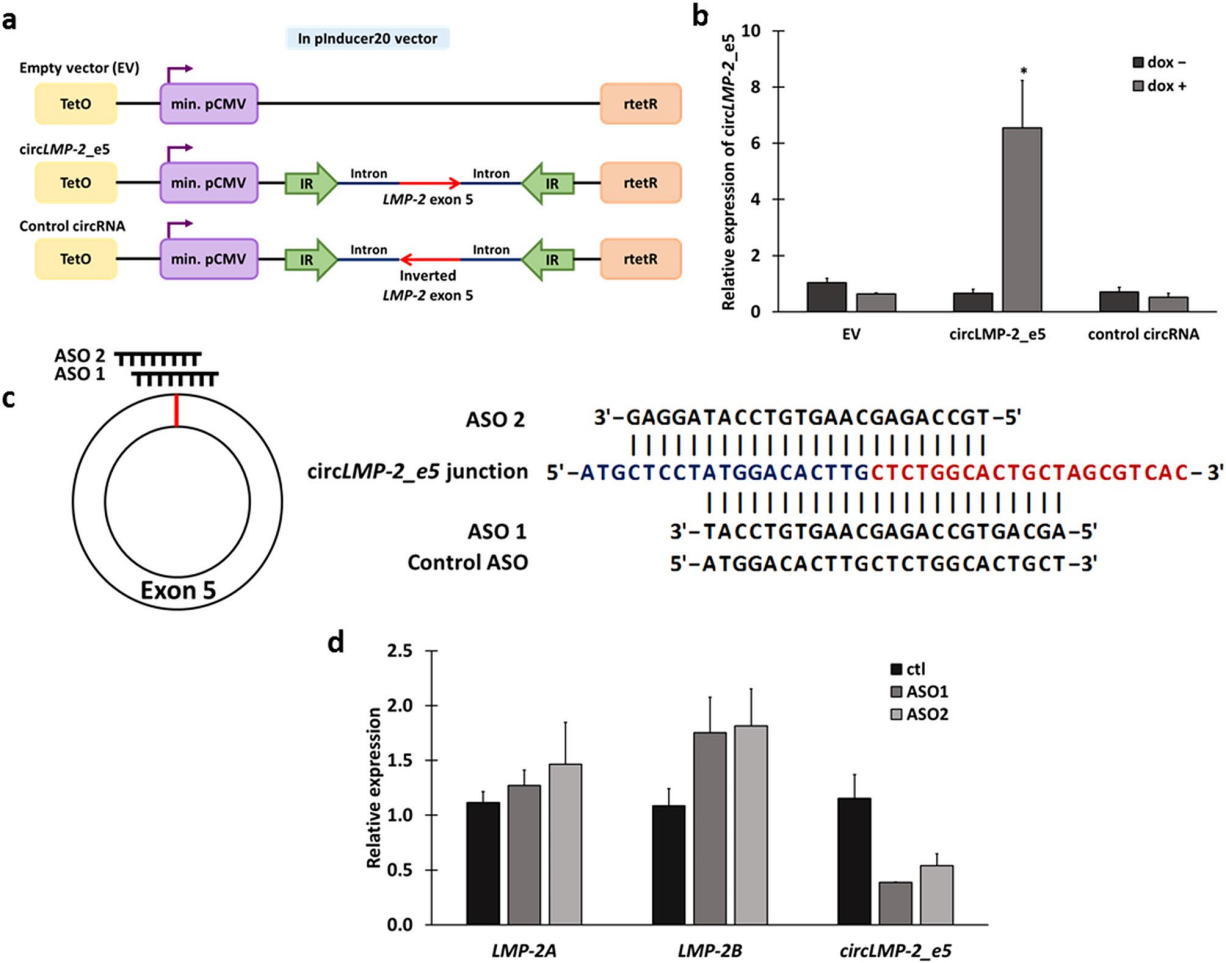
Over-expression and knockdown of circ*LMP-2*_e5 in P3HR1 and GM12878 cell lines respectively. (**a**) Schematic diagram of pInducer constructs, a Tet-on inducible lentiviral system used to induce circ*LMP-2_e5* and control circRNA (inverted *LMP-2* exon 5) over-expression. (**b**) Relative circ*LMP-2*_e5 expression with or without doxycycline induction in P3HR1 cells stably expressing EV, circ*LMP-2*_e5, and control circRNAs. Data was normalized to *ACTB* and represents the mean ± SEM of six independent experiments. (**c**) Schematic diagram of ASO targeting circ*LMP-2_e5* BSJ. Control ASO is in the sense orientation but with the same coordinate as ASO 1. (D) Relative linear *LMP-2* and circ*LMP-2_e5* expression in GM12878 cells transfected with ASO 1, ASO 2 and control ASO for 72 h. Data was normalized to *ACTB* and represents the mean ± SD of two independent experiments. [≤ 0.05 (*) and ≤ 0.01 (**)] as determined by Student’s *t*-test are indicated.

The functions of most viral circRNAs remains unknown. In order to determine the biological functions of circ*LMP-2_e5*, tet-on inducible lentiviral constructs over-expressing empty vector (EV), circ*LMP-2_e5* and inverted circ*LMP-2_e5* (control circRNA) were generated and transduced into P3HR1 cells (Fig. 5a). Upon doxycycline treatment for 72 h, circ*LMP-2_e5* expression increased significantly in P3HR1 that stably expressed circ*LMP-2_e5* compared to those without doxycycline induction and control circRNA (Fig. 5b). On the other hand, knockdown of circ*LMP-2*_*e5* was carried out in GM12878 cells by using RNase-H based antisense oligonucleotides (ASO) that target the circ*LMP-2*_*e5* BSJ (Fig. 5c). A sense strand version of circ*LMP-2_e5* ASO 1 was used as a control. ASO 1 and 2 specifically knocked-down the expression of circ*LMP-2*_*e5* without significantly affecting the linear *LMP-2* expression much (Fig. 5d). As ASO 1 has a better circ*LMP-2_e5* knockdown efficiency, therefore, it was selected for further study.

**Figure 6:**
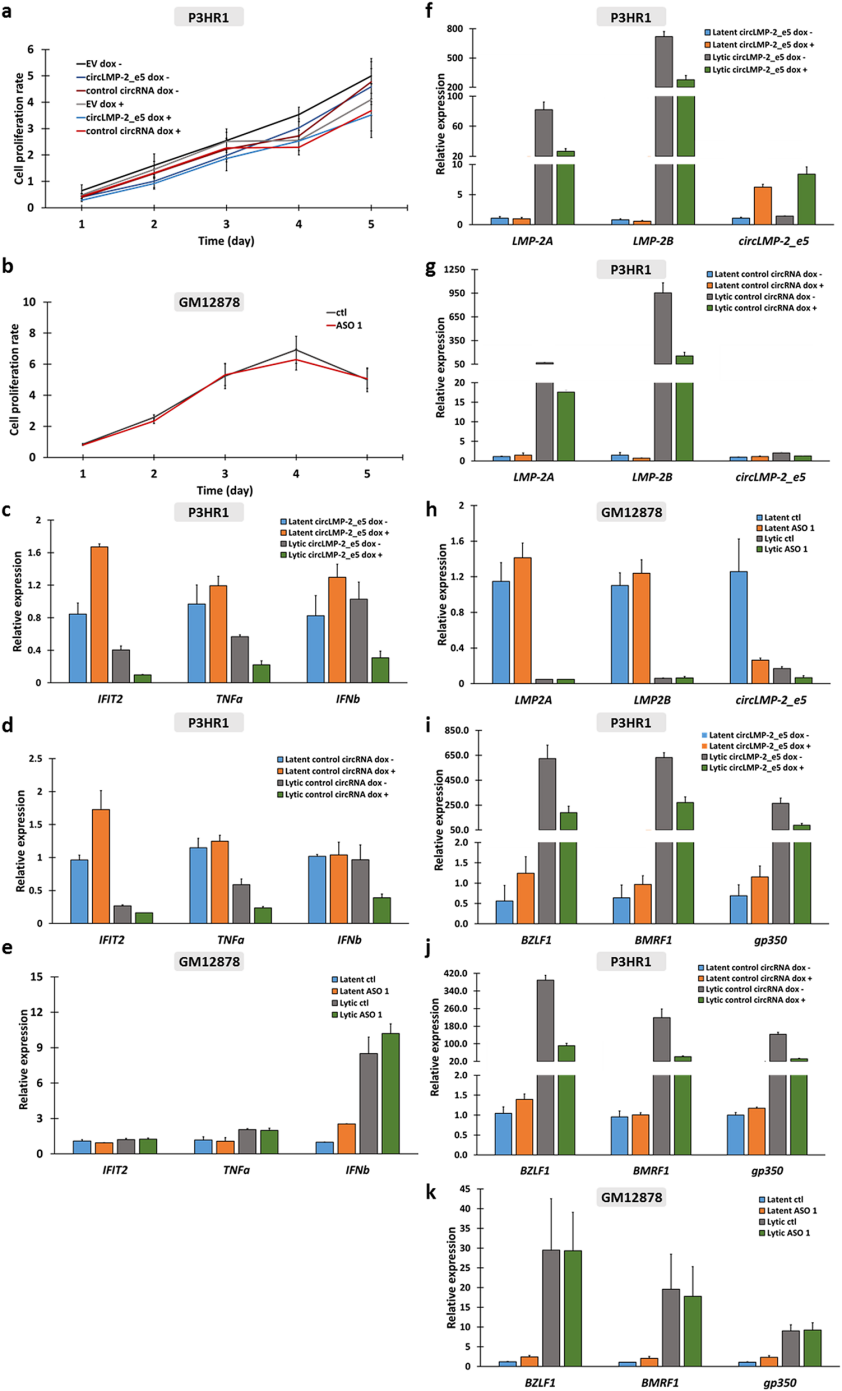
Functional characterization of circ*LMP-2*_e5. (**a**) MTT assay of P3HR1 cells induced to stably express EV, circ*LMP-2*_e5 or control circRNA for the indicated time period. Data represents the mean ± SEM of two independent experiments. (**b**) MTT assay of GM12878 cells transfected with ASO1 targeting circ*LMP-2_e5* or control ASO for the indicated time period. Data represents the mean ± SEM of two independent experiments. (**c**–**e**) Relative expression of innate immunity-related genes in P3HR1 cells stably expressing (**c**) circ*LMP-2_e5* and (**d**) control circRNA and in (**e**) GM12878 cells upon knockdown with circ*LMP-2_e5* ASO 1. (**f**–**h**) Relative expression of *LMP-2A*, *LMP-2B* and circ*LMP-2_e5* in P3HR1 cells stably expressing (f) circ*LMP-2_e5* and (**g**) control circRNA and in (**h**) GM12878 cells upon knockdown with circ*LMP-2_e5* ASO 1. (**i**–**k**) Relative expression of EBV lytic genes in P3HR1 cells stably expressing (**i**) circ*LMP-2_e5* and (**j**) control circRNA and in (**k**) GM12878 cells upon knockdown with circ*LMP-2_e5* ASO 1. Data was normalized to *RPL32/UBC* and represents the mean ± SD of at least two independent experiments except for EBV lytic gene expression data with circ*LMP-2_e5* knockdown which was normalized to *RPL32* and represents the mean ± SEM of six independent experiments.

To determine how circ*LMP-2*_*e5* expression affects the host cell, an MTT assay was performed for 4 days on P3HR1 cells induced to stably express EV, circ*LMP-2*_e5 or control circRNA and GM12878 cells with circ*LMP-2*_*e5* or control knockdown to study cell proliferation. As illustrated in Fig. 6a, P3HR1 cells with or without stable expression of EV, circ*LMP-2_e5* and control circRNA had similar proliferation rate from Day 1 to Day 5. Likewise, there was no difference in the proliferation rate of GM12878 cells with either circ*LMP-2*_*e5* or control knockdown (Fig. 6b). Together, the results suggest that circ*LMP-2_e5* does not affect the proliferation of P3HR1 and GM12878 cells. In addition, recent studies suggest viral ncRNAs could elicit host immune response^36–38^. Thus, to explore whether circ*LMP-2*_*e5* plays a role in innate immune response, the expression levels of three representative innate immunity genes (*IFIT2*, *TNFα* and *IFNβ*) were determined in P3HR1 cells with circ*LMP-2*_*e5* or control circRNA-over-expressed and in GM12878 cells with circ*LMP-2*_*e5* or control knockdown, in both latent and lytic states. P3HR1 cells with circ*LMP-2_e5* over-expressed showed a slight increase in the expression of *IFIT2*, *TNFα* and *IFNβ* in latent state, but exhibited a moderate decrease in the lytic state (Fig. 6c). However, a similar expression pattern was also observed in cells stably expressing control circRNA (Fig. 6d). Expression levels of the three innate immunity genes are also similar in GM12878 cells with circ*LMP-2*_*e5* or control knockdown in both latent and lytic states (Fig. 6e). Thus, the results suggest that circ*LMP-2_e5* is not essential for regulating innate immunity.

Recent studies have reported that circRNAs can regulate their parental genes by competing with linear splicing^39^ or promoting parental gene transcription^13^. To determine whether circ*LMP-2*_*e5* regulates linear *LMP-2* transcription, the expression levels of both *LMP-2* isoforms (*LMP-2A* and *LMP-2B*) were determined in P3HR1 cells with circ*LMP-2*_*e5* or control circRNA over-expressed, and in GM12878 cells with circ*LMP-2*_*e5* or control knockdown, in both latent and lytic states. Based on Fig. 6f and g, over-expression of circ*LMP-2_e5* and control circRNA led to a reduction in the expression of linear *LMP-2* in general. However, the effect was not sequence-specific, as over-expression of a control circRNA showed similar reduction in the expression of linear *LMP-2.* No effects on linear *LMP-2* were observed in the knockdown of ASO 1 (Fig. 6h). Therefore, circ*LMP-2_e5* is likely not involved in the transcription regulation of its cognate linear transcripts. In addition, to investigate whether circ*LMP-2*_*e5* plays a role in EBV lytic reactivation, P3HR1 cells that stably express circ*LMP-2_e5* or control circRNA as well as GM12878 cells with circ*LMP-2*_*e5* or control knockdown were induced into lytic cycle using TPA and SB for 3 days. As shown in Fig. 6i, P3HR1 cells with or without circ*LMP-2_e5* over-expression has no significant differences in the expression of EBV lytic genes (*BZLF1*, *BMRF1* and *gp350*) in the latent state. Upon entering the lytic state, expression of all three EBV lytic genes increased as expected. Over-expression of either circ*LMP-2_e5* or control circRNA led to a slight reduction of *BZLF1, BMRF1* and *gp350* (Fig. 6i–j). In the knockdown system, the expression levels of *BZLF1*, *BMRF1* and *gp350* were comparable between GM12878 cells transfected with control or ASO 1 in both latent and lytic states (Fig. 6k). Together, these results suggest that circ*LMP-2_e5* might not contribute to EBV lytic reactivation in P3HR1 and GM12878 cells.

## Discussion

In this study, we catalogued EBV-encoded circRNAs in GM12878 cells, a lymphoblastoid cell line with EBV type III latency. A total of 188 and 41 EBV putative circRNAs were detected by the psirc and find_circ algorithms, respectively, with a significant majority encoded from EBV latent genes. Circ*LMP-2_e5* appeared as the top candidate EBV circRNA from the in silico analysis according to both algorithms, and was later validated by RNase R assay and Sanger sequencing as a *bona fide* EBV circRNA. Further study of circ*LMP-2_e5* shows that it is expressed differentially across a broad range of cell lines with a different EBV latency status, with the highest expression in B cells with latency III status. Moreover, the expression pattern of circ*LMP-2*_e5 seems to mirror its cognate linear *LMP-2* gene expression and may be produced as a result of exon skipping with its circularization possibly occur without the need of *Alu* repeats or non-repetitive inverted complementary sequences in the relatively short flanking introns.. Although nucleus-retained circRNAs may modulate transcriptional processes in the host cells, our investigation into the function of circ*LMP-2_e5* in regulating cell proliferations, host innate immune response, its linear parental transcripts, and EBV lytic reactivation did not reveal significant impact on these processes.

Given that our data showed circ*LMP-2_e5* can be localized to the cytoplasm in addition to the nucleus, it is possible that circ*LMP-2*_e5 may act as a miRNA or an RNA binding protein (RBP) sponge or a template for translation. A recently identified EBV circRNA, circ*LMP-2A*, which is formed through the backsplicing of exon 5 to exon 3 of the *LMP-2A* gene, was reported to promote cancer stemness properties of EBV-associated gastric carcinoma cells through a circ*LMP-2A*/miR-3908/TRIM59/p53 axis^22^. As listed in Table S3, circ*LMP-2_e5* is predicted to contain miRNA seed sites for a total of 21 miRNAs, including 2 EBV miRNAs. All predicted miRNA seed sites are 1 or 2 in number, and are of the 7A1, 7m8 and 8-mer seed types. We used the TargetScan algorithm to predict target genes of each human miRNA and generated a circ*LMP-2_e5*–miRNA–mRNA regulatory network (Fig. S3). Based on PANTHER analysis (Fig. S4) of the top 10 miRNA-targeted genes, Toll receptor signaling pathway and CCKR signaling pathway maps are the top 2 pathways that can be potentially regulated by circ*LMP-2_e5* through miRNA sponging. However, a search for potential AGO2: miRNA: circ*LMP2-e5* interactions via interrogating publicly available AGO2 pulldown assay datasets from EBV-positive cell lines of different cell types and latencies^40–43^ did not reveal clear such interaction (data not shown). We note that these datasets only encompass the latent state, hence there is still a possibility of circ*LMP2-e5* functioning as a miRNA sponge during the lytic state. Similarly, it is possible that circ*LMP-2_e5* could interact with RBPs. Two algorithms, RBPDB^44^ and RBPmap^45^ were used to predict RBP binding sites on circ*LMP-2_e5*. A list of potential RBPs that could bind to circ*LMP-2_e5* is listed in Table S4; human_MBNL1 is the only RBP that appeared in two of the prediction sets. In addition, circRNAs can be potentially translated into protein if open reading frame can be found within it^46^. We used ATGpr software^47,48^ to predict the translational start and stop sites in circ*LMP-2_e5* but we find no evidence for circ*LMP-2_e5* being translated (Table S5). Further studies are needed to investigate if circ*LMP-2_e5* can serve as miRNA or RBP sponge, regulates other potential transcription processes, or is simply a by-product of pre-mRNA splicing.

Our data suggest that exon-skipping may give rise to circ*LMP-2-e5*. However, there is a partial inconsistency in the expression pattern of linear exon 5-skipped *LMP2* variant with the circ*LMP-2-e5* expression pattern observed in latency III cell lines. This implies that circ*LMP-2-e5* and linear exon 5-skipped *LMP-2* variant transcriptional biogenesis may be independently regulated in these cells. Skipping of *LMP-2* exon 5 is predicted to interrupt the seventh and eighth transmembrane of LMP-2. Further experiments are needed to confirm the predicted changes on LMP-2 transmembrane topology due to *LMP-2* exon 5 skipping. Nonetheless, this exon skipping might not alter the functions of LMP-2A which are dependent on the amino terminus of LMP-2A^49^. The transmembrane domains and the carboxyl terminus of LMP-2A are not involved in the initiation of primary B-lymphocyte infection, maintenance of EBV latency and lytic reactivation, or growth transformation. However, it is possible that LMP-2 exon 5 skipping would affect LMP-2B instead. An intact LMP-2B with 12 transmembrane proteins is essential for intracellular localization. N or C terminal truncations in LMP-2B proteins affecting the transmembrane regions changed its localization from intracellular perinuclear to the cell surface and retained the predicted conformation and orientation for LMP-2B^50^. Therefore, skipping of LMP-2 exon 5 may lead to changes in LMP-2B transmembrane domains 7 and 8 that would potentially alter its localization and subsequently affects its function.

Although evidence for viral circRNAs is emerging, the mechanisms for biogenesis of most viral circRNAs remain unknown. Upstream and downstream introns of the exon that formed the circRNA are known to be critical for backsplicing, and important intronic regions for backsplicing can be identified through truncated version of introns. Here, we first show that contrary to the need of long introns for circularization, circ*LMP-2_e5* is backspliced from relatively short introns. We further show that truncations of upstream (intron 4–5) and downstream (intron 5–6) introns of *LMP-2* exon 5 compromised the expression of circ*LMP-2_e5*, albeit not completely abrogated, which might be explained by the removal of predicted branchpoint sites and intron length via the lariat/exon-skipping model (Table S6 and Fig. S5). In the lariat/exon-skipping model^32^, the branchpoint downstream of a circularized exon attacks the upstream intron splice donor to form a lariat precursor during linear alternative splicing. Subsequently, the branchpoint upstream of the circularized exon attacks the downstream splice donor in the second step, positioning the 3′ end of the exon to attack its own 5′ end to form a double lariat and an exonic circRNA. It is thought that the formation of lariats enhances backsplicing catalysis by positioning the splice sites and creates a microenvironment for the splicing of circRNA. In our study, serial truncation of the flanking introns of *LMP-2* exon 5 showed that intron length may affects its circularization efficiency. A low level of backsplicing events can still occur even with a 50 bp deletion probably due to the branchpoint of the upstream intron of *LMP-2* exon 5 remains intact. In contrast, disruption of the branchpoint from the downstream intron that is located 24 bp away from its 3’ end with a 25 bp deletion leads to a drastic reduction on circ*LMP-2_e5* circularization. This result is in line with previously proposed lariat models^32^, in which the branchpoint mutants in either intron has a dramatic effect on exon circularization. Essentially, the results indicate that the first nucleophilic attack by the branchpoint in the downstream intron, which canonically plays a role in splicing to the next exon (exon 6 in this case), is important for exon-containing lariat formation to allow proper backsplicing to occur. Intriguingly, complete deletion of the downstream but not the upstream intron lead to minimal circ*LMP-2_e5* expression, suggesting the upstream intron can facilitate minimal circularization without formation of the lariat precursor. Further investigation is still required to fully elucidate the backsplicing mechanism of circ*LMP-2_e5* in particular the involvement of spliceosome and RBP.

Finally, the profiling of putative EBV circRNAs in GM12878 cells during the latent state expands the current repertoire of EBV circRNAs and serves as a useful resource for comparison of EBV circRNAs in different cell lines and diseases. The highly abundant circRNAs reported by Ungerleider et al., such as circ*EBNA_U* and circ*BHLF1* were also detected in our study albeit with lower read counts. Interestingly, the previously reported circ*RPMS1_E4_E3a* and circ*LMP-2_e8_e2* were not detected in GM12878 cells by both psirc and find_circ algorithms, which may suggest cell-type specific expression of EBV circRNAs.

## Methods

### Identification of EBV backspliced junctions

Raw RNA-seq datasets were analysed for the presence of circRNAs using the psirc (pseudo-alignment identification of circular RNAs)^23^ and find_circ^24^ algorithms. Total RNA-seq reads of subcellular (poly-A and non-poly-A) fractions of GM12878 were downloaded from GEO: GSM958730. Of these, datasets from whole-cell, cytoplasmic and nuclear fractions of GM12878 were analyzed for putative EBV circRNAs. EBV circRNA candidates were identified using the find_circ algorithm following the standard approach previously described by Memczak et al., involving alignment of reads to the genome using Bowtie, extraction of the unaligned reads, and search for noncolinear alignments based on anchors within the reads. Only reads that did not align against the hg19 version of human genome (using TopHat version 1.4.1) were used as input to the find_circ pipeline^51^. On the other hand, the putative EBV circRNAs detection part of psirc (psirc_v1.0.pl, default parameters)^23^ was run using the reference transcriptome 'chrEBV_Akata', which was taken from the Flemington Lab public repository (https://github.com/flemingtonlab/public/tree/master/annotation).

EBV circRNA candidates present with at least one read count in any library are listed in Table S1 and S2.

### Cell culture

Akata (EGFP-neo^r^ EBV–infected), P3HR1, X50-7, HK285, NPC43, C17, C666-1 and HONE1 cells were cultured in RPMI 1640 media (Gibco) supplemented with 10% FBS, 100 U/mL penicillin and streptomycin (100 mg/mL). GM12878 were cultured in RPM1 1640 media (Gibco) supplemented with 20% FBS. NPC43 and C17 media were supplemented with 4 μM Y-27632 ROCK inhibitor (Enzo Life Science), whereas C666-1 was supplemented with 1 × glutamax (Gibco). HEK293T was cultured in DMEM media supplemented with 10% FBS, 100 U/mL penicillin and streptomycin (100 mg/mL) (Gibco). All cells were cultured at 37 °C in 5% CO_2_ incubator. The cell lines used in this study were kind gifts from Professor George Tsao, University of Hong Kong, Dr. Christopher Dawson, University of Warwick, Dr. Graham Taylor, University of Birmingham, Dr. Ng Ching Ching, University of Malaya and Prof. Dr. Axel Hillmer, Genome Institute of Singapore (GIS).

### Generation of LCLs with EBV derived from NPC cases and B95.8

Tetradecanoyl-phorbol-1,3-acetate (TPA; Sigma-Aldrich) at 20 ng/mL and 3 mM Sodium butyrate (SB; Sigma-Aldrich) were used to induce EBV virion production from cells according to established methods^52,53^. Two million isolated peripheral blood mononuclear cells (PBMCs) were infected with 1.0 × 10^4^ copies of EBV. LCLs were formed after two weeks from PBMCs infected with B95-8-derived EBV but took longer time (3 to more than 12 weeks) for PBMCs infected with NPC-derived EBVs. Mock control did not result in establishment of LCLs. The LCLs were characterized by EBER in situ hybridization and immunocytochemistry for EBV BZLF1.

### Induction of EBV lytic reactivation

Suspension and adherent cells were lytic reactivated at a cell density of 5 × 10^5^/mL and 2.5 × 10^5^/mL respectively except for C17 which was seeded at the density of 4 × 10^5^/mL. During lytic reactivation, ROCK inhibitor was not included in the media. Akata was reactivated using 10 μg/mL of Goat (Fab)_2_ fragment anti-human immunoglobulin G (IgG) (MP Biomedicals) for 24 h, C17 was reactivated by transfection with 1 μg BZLF1-expressing plasmid p509 (kindly provided by Prof. Wolfgang Hammerschmidt, German Research Center for Environmental Health, Munich, Germany) for 72 h using X-tremeGene HP DNA transfection reagent (Roche), NPC43 was reactivated using 40 ng/mL TPA (Sigma-Aldrich) and 0.3 mM SB (Sigma-Aldrich) for 48 h; GM12878 was reactivated with 200 ng/mL TPA and 1.2 mM SB while the rest of the cell lines were induced into lytic state using 50 ng/mL TPA and 0.3 mM SB for 72 h. All treated and untreated cells were harvested and subjected to total RNA extraction.

### Quantitative RT-PCR

Total RNA was isolated with Macherey Nagel NucleoSpin® RNA kit, followed by DNase I treatment. Random hexamer- or primer specific-converted cDNA was synthesized using M-MuLV reverse transcriptase (NEB) according to manufacturer’s protocol. Quantitative RT-PCR for linear and circular RNA gene expression were performed with gene specific convergent and divergent primers (Supplementary Table S7) respectively using 2 × Kappa SYBR Green PCR Master mix or 2 × Kappa Probe Fast Master Mix (for Taqman analysis) according to the manufacturer’s protocol and run on a Bio-Rad Connect Real-Time PCR System. For newly-generated LCLs, Qiagen AllPrep DNA/RNA/miRNA Mini Kit was used for RNA isolation, followed by cDNA conversion using SuperScript IV VILO Master Mix according to the manufacturer’s protocol. Linear and circular RNA gene expression were quantified using Applied Biosystem TaqMan™ Fast Advanced Master Mix in Applied Biosystems™ 7500 Real-Time PCR System. Expression fold change was normalized by respective housekeeping gene and calculated using the comparative Ct method (2^−∆∆Ct^).

### RNase R assay

Twenty microgram of total RNA was treated with or without 20 units of RNase R (Epicentre) at 37 °C for 2 h followed by DNase I (NEB) treatment. RNA was then purified using the lithium chloride method and eluted in 30 μL of RNase-free water^54^. The cDNA was synthesized as mentioned above and PCR was performed using specific divergent primers to validate the candidate EBV circRNAs.

### Subcellular fractionation and RNA extraction

Subcellular fractionation protocol was adapted and modified from a previously described protocol^55^. Briefly, GM12878 cells were resuspended in a hypotonic buffer (10 mM Tris (pH 7.5), 10 mM KCl, 1.5 mM MgCl_2_, 0.5 mM DTT, 0.075% NP-40, and 2 mM Ribonucleoside vanadyl complexes) and incubated on ice for 5 min, followed by 10 min centrifugation at 500×*g*, 4 °C. Supernatant was collected as cytoplasmic fraction and the pellet was washed 3 times with hypotonic buffer. The cytoplasmic fraction was subjected to 1 mL of RNA precipitation solution (RPS; 0.15 M sodium acetate (pH 5.5) in ethanol) and incubated at − 20 °C for 1 h. The cytoplasmic fraction in RPS was vortexed and centrifuged at 18,000×*g* for 15 min, 4 °C. The supernatant was removed and the pellet was rinsed with 70% (v/v) ice-cold ethanol. Approximately 1 mL of Trizol was added to the semi-dry nuclear and cytoplasmic pellets followed by addition of 10 μL of 0.5 M EDTA. Both fractions were then heated at 65 °C until the pellet dissolved. Total RNA isolation from both cytoplasmic and nuclear fractions were then continued with the Trizol RNA extraction method modified from the manufacturer’s protocol. Relative expression of each genes was calculated based on the equation:

1 $$Relative \, expression \, in \, cytoplasm=\frac{{2}^{\left({Cq}_{cyto}-{Cq}_{nuc}\right)}}{RNA \, ratio}$$

2 $$Relative \, expression \, in \, nucleus=\frac{{2}^{\left({Cq}_{nuc}-{Cq}_{cyto}\right)}}{RNA \, ratio}$$

RNA ratio is the ratio of cytoplasmic RNA concentration to nuclear RNA concentration eluted in similar amount of water. All gene expression were calculated using formula (1), except for *MALAT1* which was calculated using formula (2).

### Truncated circ*LMP-2_e5* constructs and transfection

EBV genomic DNA was used as the template for the amplification of full length and truncated circ*LMP-2_e5* regions. Primers used were flanked with either EcoRI or XhoI. The full length and truncated circ*LMP-2_e5* fragments were cloned into pcDNA3 vector. All constructs generated were verified by Sanger DNA sequencing. Each construct was transfected into HONE1 cells using Roche X-tremeGENE HP transfection reagent for 48 h before proceeding with total RNA isolation.

### Inducible circ*LMP-2_e5* construct and its ectopic expression

An inducible system—pInducer20-circ*LMP-2_e5* plasmid was constructed to over-express circ*LMP-2_e5* in P3HR1 cells. pInducer20 (Addgene) was used as the lentiviral vector backbone for the generation of a Tet-on inducible system^56^. *LMP-2* exon 5 with flanking upstream and downstream introns was first cloned into pcDNA3 and then sub-cloned into a region flanked by inverted repeats in circR plasmid^57^ which is a kind gift from Professor Gregory Goodall. Then, circ*LMP-2_e5* sequence flanked with inverted repeats was sub-cloned into pInducer20 vector in place of *ccdB* gene. A similar length of circRNA negative control containing the inverted version of circ*LMP-2_e5* (named as control circRNA) was also constructed in the same manner. Circ*LMP-2_e5* and control circRNA sequences were flanked with inverted repeats to enhance circRNA circularization for high-copy-number circRNA expression. The plasmids were subsequently verified by Sanger DNA sequencing. Third generation lentiviral system was utilized for the lentivirus production by transfecting HEK293T cells with the lentiviral vectors including the construct of interest and lentiviral packaging plasmids (RRE, REV, VsVG) using calcium phosphate transfection method. The viral supernatant collected after 48 h of transfection was used to transduce P3HR1 cells in the presence of 1 µg/mL polybrene and selected in 750 µg/mL G418 (Cayman). Ectopic expression of empty vector (EV), circ*LMP-2_e5* and control circRNA in P3HR1 cells was induced with 0.5 μg/mL doxycycline for 72 h.

### ASO transfection

ASO 1 (5′-mA^*^mG^*^mC^*^mA^*^mG^*^mU^*^G^*^C^*^C^*^A^*^G^*^A^*^G^*^C^*^A^*^A^*^G^*^T^*^mG^*^mU^*^mC^*^mC^*^mA^*^mU-3′), ASO 2 (5′-mU^*^mG^*^mC^*^mC^*^mA^*^mG^*^A^*^G^*^C^*^A^*^A^*^G^*^T^*^G^*^T^*^C^*^C^*^A^*^mU^*^mA^*^mG^*^mG^*^mA^*^mG-3′) and control (5′-mA^*^mU^*^mG^*^mG^*^mA^*^mC^*^A^*^C^*^T^*^T^*^G^*^C^*^T^*^C^*^T^*^G^*^G^*^C^*^mA^*^mC^*^mU^*^mG^*^mC^*^mU-3′) were synthesized by IDT technologies, and 100 nM of ASOs were transfected into GM12878 cells with Promega FuGENE HD Transfection Reagent according to the manufacturer’s protocol. To maximize knockdown efficiency, ASO transfection was repeated 24 h after the initial transfection.

### MTT assay

Two thousand P3HR1 stable cells per well were seeded in a 96-well round bottom plate. Ectopic expression of circ*LMP-2_e5* or control circRNAs was induced with 0.5 μg/mL of doxycycline. Cells seeded 24 h later were used as Day 1 and doxycycline was replenished at Day 3. Five thousand GM12878 cells with ASO 1 or control ASO double transfections per well were seeded in 96-well round bottom plate. GM12878 cells seeded 24 h later were used as Day 1. At 24 h intervals, 10 µL (2 mg/mL) MTT solution was added to each well of a plate followed by incubation for 2 h at 37 °C in a CO_2_ incubator. The plates were then centrifuged at 2000 rpm for 5 min and the supernatants were carefully removed. The formazan reaction product was then dissolved in 100 µL DMSO and the absorbance at 570 nm was measured with a M200 PRO microplate reader (Tecan).

## Supplementary Information

Supplementary Information 1.

Supplementary Information 2.
