# Supplementary material for: Identification and characterization of a novel Epstein-Barr Virus-encoded circular RNA from LMP-2 Gene
Source: Sci Rep. 2021 Jul 13;11:14392. doi: 10.1038/s41598-021-93781-w (PMC8277822; doi:10.1038/s41598-021-93781-w)
Supplement: Supplementary file 2 — Supplementary Information 2. [file 41598_2021_93781_MOESM2_ESM.docx]

**Supplementary Materials**


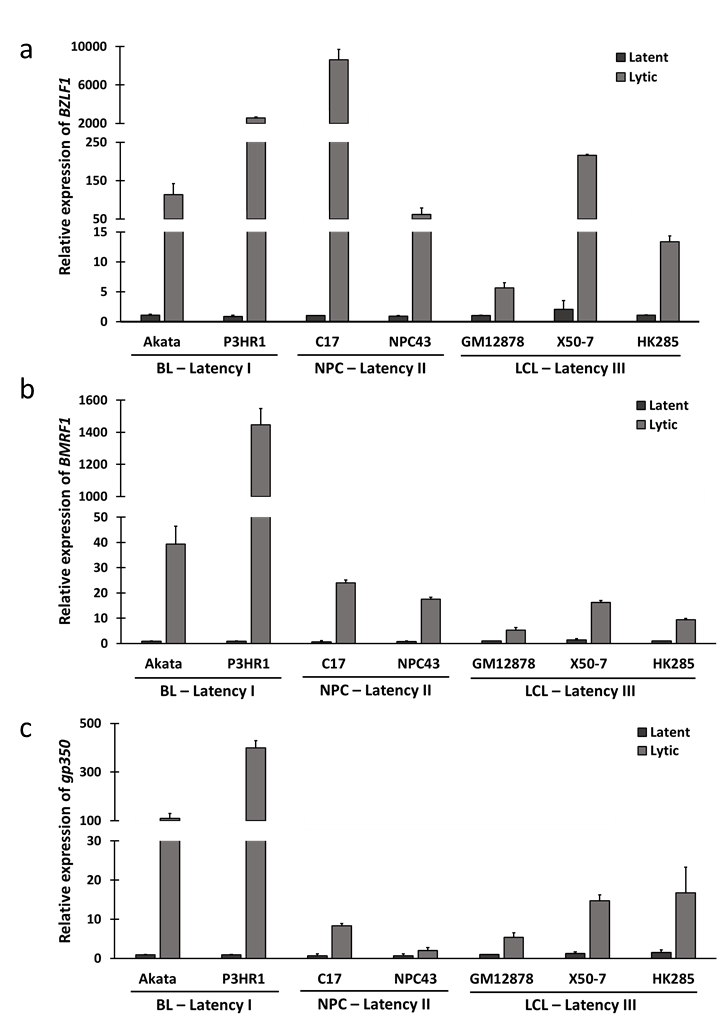


**Figure S1. RT-qPCR analysis of EBV lytic genes in various EBV-positive cell lines.** Expression of (a) *BZLF1*, (b) *BMRF1* and (c) *gp350* in EBV-positive cell lines with different latency programs in latent and lytic states.


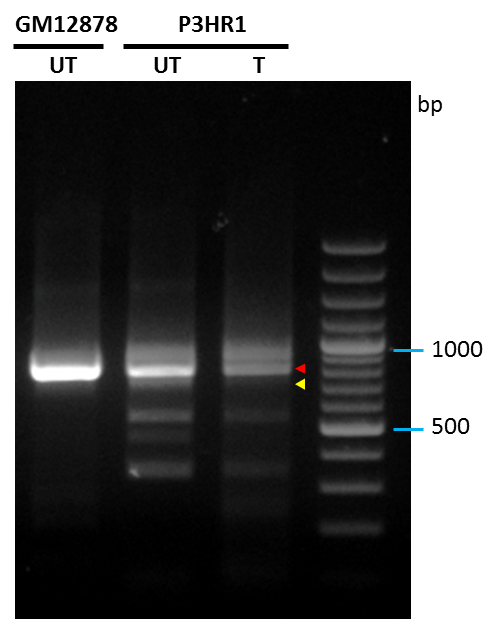


**Figure S2**. Linear *LMP-2* (red arrow) and *LMP-2* splice variant with *LMP-2* exon 5-skipped (yellow arrow) were detected in EBV-positive cell lines, GM12878 and P3HR1in latent (UT) state, as well as in P3HR1 which undergo lytic reactivation (T) using 50 ng/mL TPA and 0.3 mM SB. Full-length gel image is shown in Fig. S6.

**Table S3**. miRNA binding site and its binding type.

|  | **miRNA seed sites** | | |
| --- | --- | --- | --- |
|  | **7A1** | **7m8** | **8** |
| hsa-miR-1252-5p | 0 | 2 | 0 |
| hsa-miR-6770-5p | 0 | 0 | 2 |
| hsa-miR-8063 | 0 | 2 | 0 |
| hsa-miR-28-5p | 2 | 0 | 0 |
| hsa-miR-12120 | 0 | 2 | 0 |
| hsa-miR-3912-5p | 0 | 2 | 0 |
| hsa-miR-1253 | 2 | 0 | 0 |
| hsa-miR-4276 | 0 | 2 | 0 |
| hsa-miR-3139 | 2 | 0 | 0 |
| hsa-miR-5190 | 0 | 0 | 2 |
| hsa-miR-6513-3p | 0 | 2 | 0 |
| hsa-miR-4519 | 0 | 0 | 2 |
| hsa-miR-5094 | 0 | 2 | 0 |
| hsa-miR-708-5p | 2 | 0 | 0 |
| hsa-miR-4274 | 2 | 0 | 0 |
| hsa-miR-648 | 0 | 2 | 0 |
| hsa-miR-595 | 0 | 0 | 2 |
| hsa-miR-621 | 0 | 2 | 0 |
| hsa-miR-6501-3p | 0 | 1 | 0 |
| ebv-miR-BART18-5p | 2 | 0 | 0 |
| ebv-miR-BART2-3p | 2 | 0 | 0 |


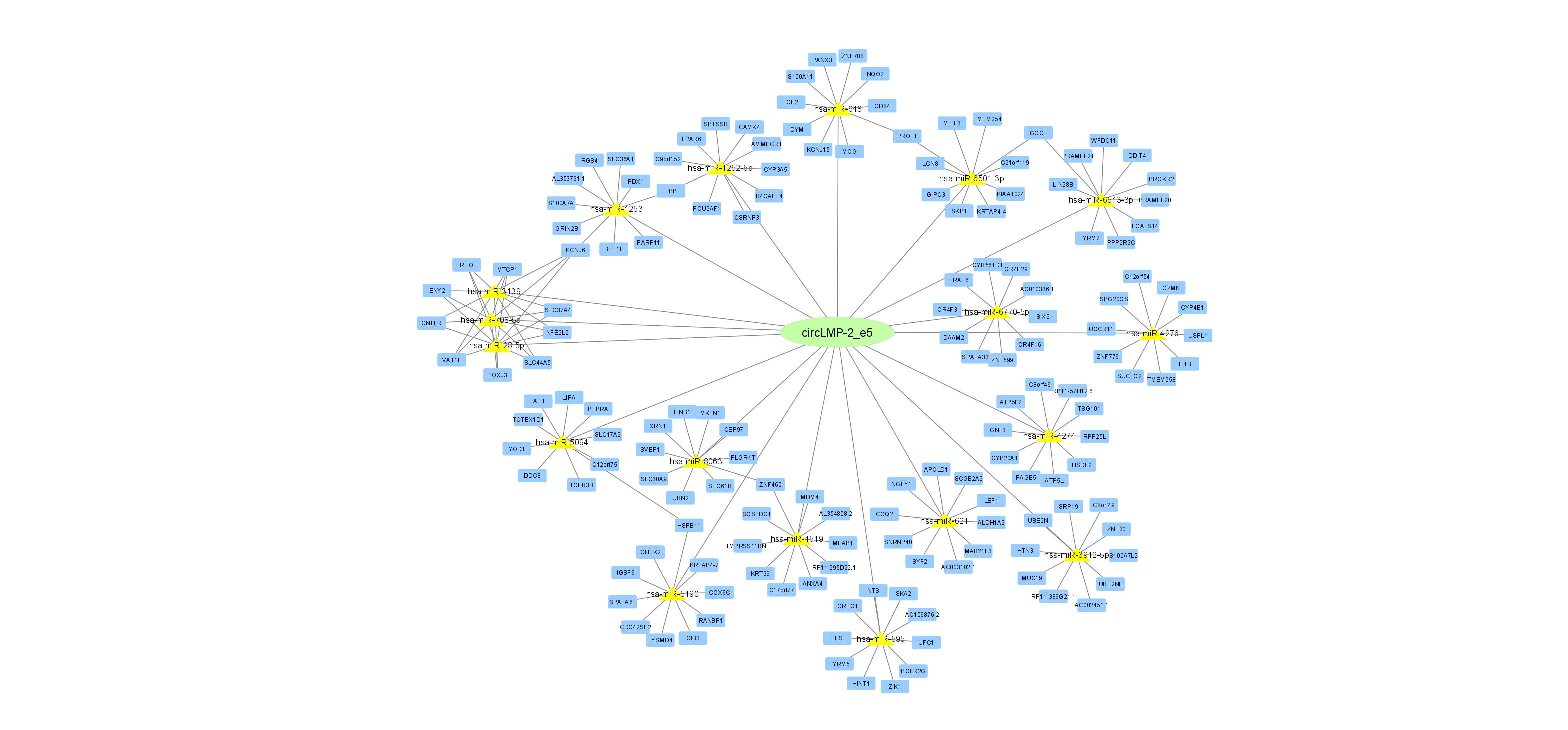


**Figure S3**. circRNA–miRNA–mRNA regulatory network. The network consisting of circ*LMP-2_e5,* 18 human miRNAs and 10 respective miRNA-targeted genes was generated by Cytoscape 3.8.2. TargetScan was applied to predict the target genes of each miRNA.


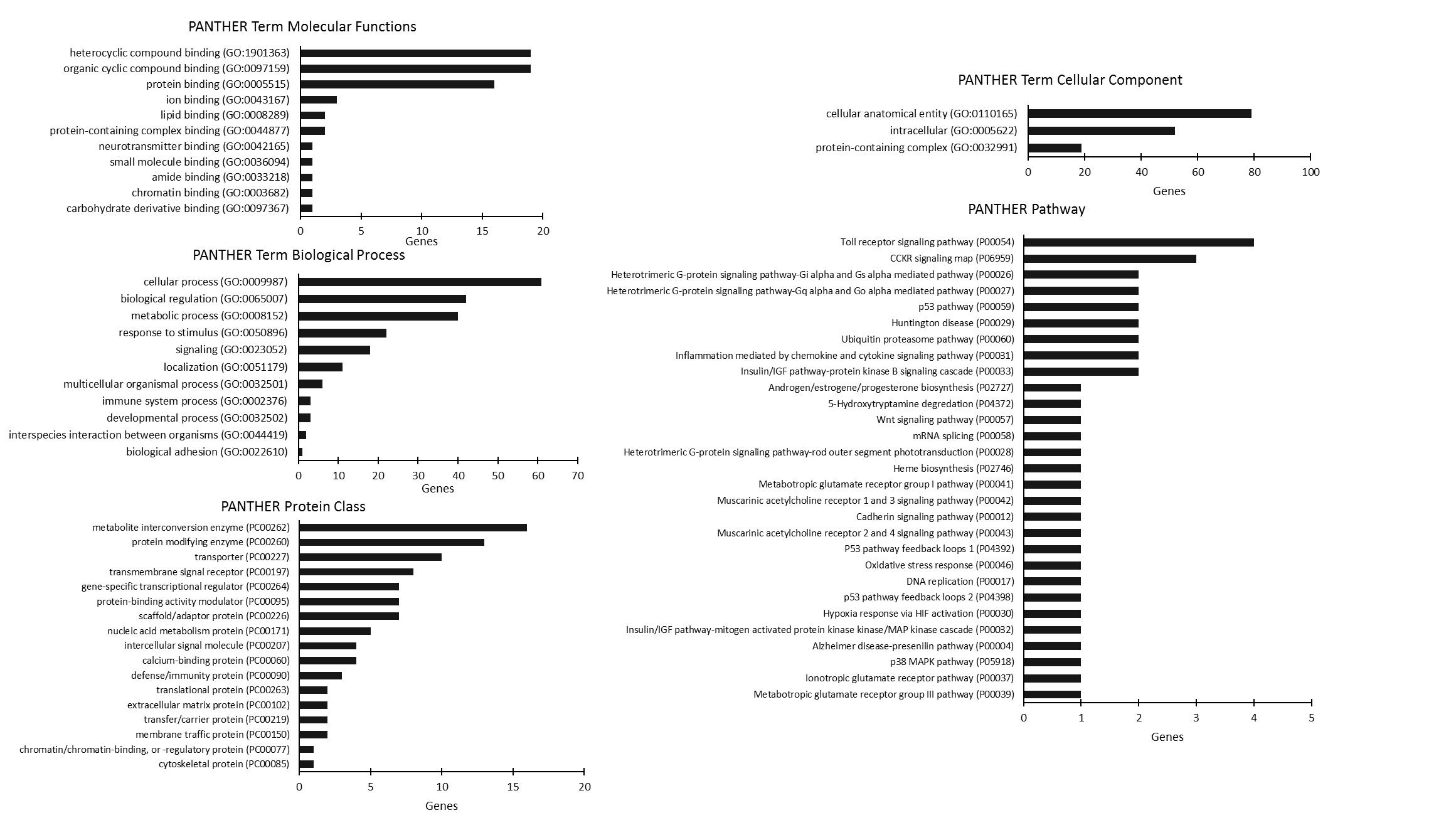
**Figure S4**: PANTHER analysis of genes that potentially involved in circ*LMP-2_e5* ceRNA network.

**Table S4**. RBP prediction using RBPDB and RBPmap software. Human_MBNL1 is the only RBP that appeared in 2 software.

|  | **Software used** | |
| --- | --- | --- |
|  | **RBPDB** | **RBP map** |
| human_MATR3 |  | √ |
| human_MBNL1 | √ | √ |
| human_RBM41 |  | √ |
| human_RBM45 |  |  |
| human_SNRPA |  | √ |
| human_SRSF1 | √ |  |
| human_SRSF7 |  |  |
| human_ZC3H10 |  |  |
| human_YTHDC1 | √ |  |
| human_EIF4B | √ |  |
| human_ELAVL1 | √ |  |
| human_KHDRBS3 | √ |  |
| human_SRSF2 |  | √ |
| human_SRSF3 |  | √ |
| human_CUG-BP |  | √ |
| human_SRSF5 |  | √ |

**Table S5**. Prediction of translational start and stop sites using ATGpr software.

| # | Reliability | Frame | Identity to Kozak Rule A/GXXATGG | Start (bp) | Stop codon |
| --- | --- | --- | --- | --- | --- |
| 1 | 0.04 | 3 | AXXATGt | 51 | No |
| 2 | 0.04 | 3 | cXXATGc | 63 | No |
| 3 | 0.04 | 2 | cXXATGG | 71 | No |

**Table S6**. Prediction of branchpoints found in upstream and downstream introns of *LMP-2* exon 5 by SVM-BPfinder.

**
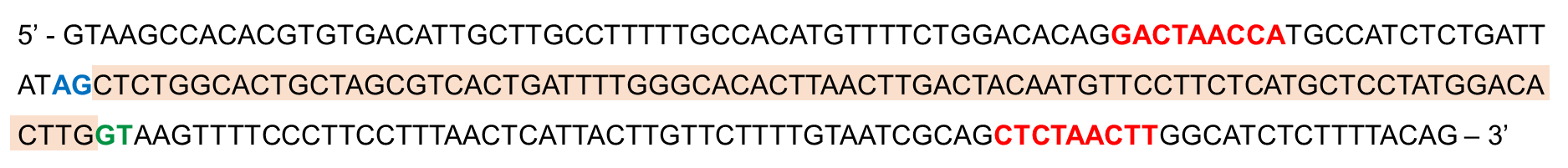
**

**Figure S5**. **Sequence of LMP-2 exon 5 (highlighted in pink) flanked with upstream and downstream introns that is essential for circ*LMP-2_e5* biogenesis.** Potential branchpoints in both upstream and downstream introns (in red) were predicted by SVM-BPfinder. Canonical splice sites are also labeled in blue (splice acceptor) and green (splice donor).


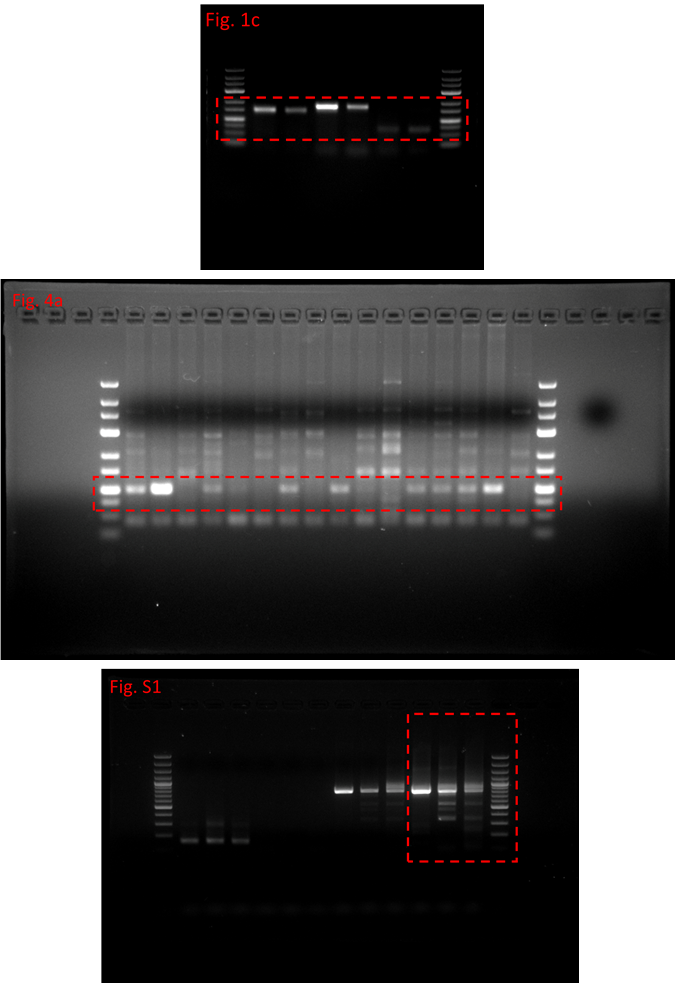


**Figure S6. Original full image of agarose gels with the corresponding figure numbers labeled on top left corner.** Red dotted rectangles refers to cropped areas.
